# Charges for Professional Service

**Published:** 1892-08

**Authors:** 


### CHARGES FOR PROFESSIONAL SERVICE.

The amount charged for services is one of the most important factors that help to make or mar the fortunes of the modern practitioner of dentistry. This is especially the case with the beginner. Let him be ever so neat, gentlemanly, and thorough in his work, and charge four or five dollars an hour, he will spend most of his time idly, sighing for the patients that never come. While across the way he may see an untidy person constantly busy over an ever-increasing crowd of patients.

The cause of this most unfortunate state of affairs is only too evident. The experienced dentist is not paid merely for excavating a tooth, or for materials placed therein. This fee remunerates for skill, for delicacy of manipulation, for the perfect confidence which he inspires in his patient, and for many other desirable qualities which years of practice alone can give.

If this be true, the failure of the beginner to build up a practice while charging the same price as the older dentist is only what might be expected. Until he has a certain amount of experience outside of the dental college he should keep the fees reduced to such a point that patients will come to him in numbers.

One of the most successful practitioners of Philadelphia, in speaking of his early professional life, said, "I made it a rule to be always busy. If I could not fill my time at two dollars an hour, I charged one dollar. If one dollar could not be paid, rather than be idle I worked simply for the cost of the materials. Thus, my hand gained skill, my experience enlarged, and the number of my patients increased. True, the low-priced patients, in themselves, did not bring much money, but often, very often, they were the means of sending me their friends, who paid liberally."

The man whose practice consists of a few rich families, charged exorbitantly, occupies a humiliating position, as he is necessarily entirely dependent upon them for his material existence.

A dentist to be independent should have a practice so large that the loss of three or four of his most wealthy patients should be a matter of little moment. To bring about this desirable state of affairs he should be sure to give a liberal return for money received.

But, it has been said by beginners, " If we start with our prices too low, how shall we be able to raise them?"

The answer to this is clear and simple.

If a dentist has eight hundred patients, all of whom pay two dollars an hour for services, he can first raise the prices to new-comers, and later, as confidence in his position is established, he can extend the advance throughout the entire practice.

Many established practitioners regard low prices in beginners as a fault, as an attempt to undermine the average scale supposed to be charged by the profession at large. Guided by their protests, many a conscientious beginner fails to collect a practice, where one without regard for dental ethics succeeds.

A professional man is paid quite as much for the manner of performing the work as for the work itself. Leading actors like Booth or Jefferson are not disturbed because large numbers of professionals work for a small sum nightly. This fact in no wise jeopardizes their large incomes. So long as their work maintains its high excellence they need not fear competition.

There are hundreds of teeth extracted to-day because the people cannot afford to pay the ordinary charges. This is so much work lost to the profession at large, so much actual money thrown away, so much benefit taken from the community. There are many graduates who would willingly perform this labor for very moderate prices if they could get it. But they are too apt to drive it away by indiscretion.

Almost the first questions a young practitioner asks is, " What shall I charge? What do you charge?" These two questions many times have caused him years of sorrow and hopeless longing for the clientele that never comes.

If he had commenced with the idea of doing a great deal of work for a little money, his skill would have increased, his experience enlarged, his reputation spread. Patients would have flocked to him as to the one person whom they would permit to fill their teeth, and then, independent of the whims or idiosyncrasies of the few, he could have charged fair prices with a feeling of security in the rapid development of his reputation and the enlargement of his bank account.

